# The Relationship Between Limited English Proficiency and Outcomes in Stroke Prevention, Management, and Rehabilitation: A Systematic Review

**DOI:** 10.3389/fneur.2022.790553

**Published:** 2022-02-03

**Authors:** Jeffrey R. Clark, Nathan A. Shlobin, Ayush Batra, Eric M. Liotta

**Affiliations:** Department of Neurology, Feinberg School of Medicine, Northwestern University, Chicago, IL, United States

**Keywords:** stroke, limited English proficiency, health literacy, healthcare disparities, communication barriers

## Abstract

**Introduction:**

Individuals with limited English proficiency (LEP) face structural challenges to communication in English-speaking healthcare environments. We performed a systematic review to characterize the relationship between LEP and outcomes in stroke prevention, management, and recovery.

**Methods:**

A systematic review was conducted using the PubMed, Embase, Scopus, and Web of Science databases. Titles and abstracts from articles identified were read and selected for full text review. Studies meeting inclusion criteria were reviewed in full for study design, aim, and outcomes.

**Results:**

Of 891 unique articles, 20 were included. Eleven articles did not provide information about interpreter availability or usage, limiting the ability to draw conclusions about the effect of LEP on measured outcomes in these studies. Overall, studies demonstrated that English proficiency is associated with better outcomes in preventive aspects of stroke care such as stroke symptom awareness, anticoagulation maintenance, and knowledge of warfarin indication. Some acute stroke care metrics were independent of English proficiency in seven studies while other evidence showed associations between interpreter requirement and quality of inpatient care received. LEP and English-proficient groups show similar mortality despite greater lengths of stay and greater proportions of care in dedicated stroke units for LEP patients. Post-stroke quality of life can be worse for those with LEP, and language barriers can negatively impact patient and provider experiences of rehabilitation.

**Conclusions:**

Stroke patients with LEP face barriers to equitable care at multiple stages. While some studies demonstrate worse outcomes for LEP patients, equitable care was shown in multiple studies frequently in the setting of a high degree of interpreter availability. Patients with LEP will benefit from tailored education regarding stroke symptom recognition and medication regimens, and from provision of translated written educational material. Inequities in inpatient care and rehabilitation exist despite similar mortality rates in four studies. Future studies should report interpreter availability and usage within LEP groups and whether patient interactions were language-concordant or discordant in order to allow for more generalizable and reliable conclusions.

## Introduction

Individuals who self-report that they speak English less than “very well” can be considered to have limited English proficiency (LEP) (1) and face structural challenges to communication in English-speaking healthcare environments, typically relying on professional medical interpreters (PMIs), family, or multilingual providers to surmount language barriers which can jeopardize care (1–3). LEP is associated with delays in seeking medical attention (4), negative impacts on patient satisfaction (5), and lower utilization rates of preventive care (6, 7). Healthcare in stroke, the treatment of which is time-sensitive and depends on eliciting descriptions of symptoms and times of onset from patients, is particularly vulnerable to the effects of language barriers.

While PMI services have been shown to improve outcomes, patient satisfaction, and efficiency of care delivery in a variety of settings (8), the extent and circumstances of their involvement vary, and associations between their involvement and the quality of care received by stroke patients remain unclear (9). There has been little synthesis of the effects of LEP and utilization of PMIs before, during, and after the inpatient phase of the care of stroke patients. We performed a systematic review to identify differences in outcomes in stroke care prevention, management, and recovery between individuals with and without English proficiency in English-predominant healthcare settings. Our findings may assist healthcare providers to pursue equitable care for LEP individuals at risk for, afflicted by, and recovering from stroke.

## Methods

A systematic review was conducted in accordance with the Preferred Reporting Items for Systematic Review and Meta-Analysis (PRISMA) using the population, intervention, comparator, outcome, and study designs (PICOS) structure (10). Our population of interest was individuals at risk for, affected by, or recovering from stroke with a specified degree of English proficiency and necessarily in English-predominant healthcare settings. Study inclusion required that prespecified outcomes were compared between groups of differing English proficiency, and interventions were not relevant as included studies were observational. Prespecified outcomes included rates of patient usage of and adherence to preventive stroke care regimens, routinely used metrics of acute and inpatient stroke care quality and efficiency such as door-to-needle (DTN) time, hospital length of stay (LOS), adverse events, mortality, discharge disposition, neurological status/functional independence at discharge, and post-stroke quality of life. PubMed MEDLINE (National Library of Medicine), Embase (Elsevier), Scopus (Elsevier) and Web of Science (Clarivate Analytics) were searched in September 2021 from inception without restrictions on date, language, or publication type. Our search strategy utilized MeSH heading terms and user-defined terms, and is provided in Supplementary Table 1. No protocol was registered, and no external funding was received for this study. As we performed a systematic review of existing literature and no primary data were collected, institutional review approval was not required.

Articles selected for full text review were included if they met the following prespecified criteria: published in or translated into English, full-length journal article with full text available, providing content pertinent to stroke and LEP, and discussing the prespecified outcomes above. Duplicate publications were removed, and all remaining publications were screened for relevance based on title and abstract by two study authors with a third author consulted for disagreements as necessary. All articles selected for final inclusion were reviewed for study design, subject characteristics, country of origin, definitions used to categorize study subjects, and reported outcomes. Quality of included studies was graded in accordance with the framework created by Shadish et al. (11). Study grades are presented in Table 1. Grade E studies were excluded from this review. The risk of bias for each study was determined based on the Newcastle-Ottawa Quality Assessment tool and is presented in Table 2 (12). Heterogeneity of study designs, outcomes, and participants precluded a meta-analysis.

**Table 1:** Grading of study design quality (11).

**Grade**	**Design**
AA	Systematic review or meta-analysis of RCTs
A	Systematic review or meta-analysis of non-RCTs
	RCT or cluster RCT
B	Systematic review or meta-analysis of controlled studies without a pretest or uncontrolled study with a pretest
	Non-RCT
	Controlled before-&-after study
	Retrospective or prospective cohort study
	Interrupted time series
	Case-control study
C	Systematic review or meta-analysis of cross-sectional studies
	Uncontrolled before-&-after study
D	Cross-sectional study
E	Case studies, case reports, narrative reviews

*RCT, randomized controlled trial*.

**Table 2:** Studies included in the systematic review.

**References**	**Study design and size**	**Study design quality (11)**	**Article quality** **(12)**	**Country**	**Basis of LEP or language preference**	**Language-based participant exclusions**	**Availability and quality of PMIs**	**Key findings**
Anderson et al. (31)	Retrospective cohort study *N* = 928	B	Good	USA	Primary language was defined by self-report as the language in which the patient preferred to communicate.	No language-based exclusions were made.	Spanish and Vietnamese available in person in the ED during business hours, telephone PMIs available 24/7. Interpretation quality was unknown/not stated.	There were no significant differences between English, Spanish, or other language speakers in quality metrics such as rate of receiving thrombolysis, DTI time, DTN time, and hospital LOS, nor were there differences in mortality.
Bhandari et al. (15)	Retrospective cohort study *N* = 864	B	Good	USA	Primary language was defined by self-report as the language in which the patient preferred to communicate.	No language-based exclusions were made.	Quality, availability, and rates of PMI usage were unknown/not stated.	TTR was 7.2% lower for Spanish-speaking Hispanic patients than for English-speaking Hispanic patients (*p* <0.05) despite intensity of care being indistinguishable across all groups.
Davies et al. (28)	Retrospective case-control study *N* = 160	B	Good	Australia	LEP was defined as requiring PMI services.	No language-based exclusions were made.	PMIs were available in-house and PMIs underwent cultural competence training. Interpretation quality was unknown/not stated.	Rehabilitation outcomes and time spent with therapists did not differ between LEP and English-proficient groups, however, within the LEP group, patients receiving higher levels of PMI services made greater improvements in FIM efficiency.
DuBard et al. (13)	Cross-sectional study *N* = 25,426	D	Good	USA	Primary language was defined as the language in which the survey was administered and answered.	No language-based exclusions were made.	Spanish-speaking respondents were surveyed by a Spanish-speaking interviewer. Interpretation quality was unknown/not stated.	Spanish-speaking Hispanics were less likely than English-speaking Hispanic, non-Hispanic white, and non-Hispanic black patients to correctly identify stroke symptoms (18% of respondents vs. 31, 50, and 41%, respectively, *p* <0.001).
Erfe et al. (21)	Retrospective cohort study *N* = 3,295	B	Good	USA	Primary language was defined by self-report as the language in which the patient preferred to receive medical information.	No language-based exclusions were made.	PMIs were available 24/7 with a mixture of in-person, phone, or video. Interpretation quality was unknown/not stated.	After adjusting for socioeconomic factors, age, sex, and initial NIHSS, likelihood of receiving IV thrombolysis did not differ for patients who preferred a language other than English.
Erfe et al. (24)	Retrospective cohort study *N* = 259	B	Good	USA	Groups were defined based on receiving PMI services or not, within a population of non-English preferring patients as defined by self-report.	Included only non-English preferring patients.	PMIs were available 24/7 with a mixture of in-person, phone, or video. Interpretation quality was unknown/not stated.	Non-English-preferring patients who did not receive a PMI were less likely to receive defect-free care than patients who did receive PMI services (61.5 vs. 73.9%, *p* = 0.04, adjusted model OR 0.49, 95% CI 0.25–0.94), where defect-free care represented receipt of all treatment measures for which a patient was eligible.
Fang et al. (14)	Cross-sectional study *N* = 183	D	Good	USA	Primary language was defined by self-report as the language in which the patient preferred to communicate.	Included only English, Spanish, Mandarin, or Cantonese speakers.	Trained multilingual study personnel were provided to each patient.	Not speaking English was independently associated with discordant descriptions of warfarin indication.
Fryer et al. (27)	Cross-sectional study *N* = 156	D	Poor	Australia	LEP was defined as requiring PMI services.	No language-based exclusions were made.	Quality, availability, and rates of PMI usage were unknown/not stated.	Patients requiring PMI services post-stroke needed more assistance with ADLs, had lower activity levels and rates of exercise, had slower gait speed and TUG, and utilized fewer home health services.
Fryer et al. (29)	Cross-sectional study *N* = 14	D	Poor	Australia	Primary language was defined by self-report as the language in which the patient preferred to communicate.	No language-based exclusions were made.	PMIs with study-specific training were provided to each patient unless the patient declined. Interpretation quality was unknown/not stated.	Patients requiring PMI services post-stroke reported a variety of difficulties in rehabilitation pertaining to communication and active involvement in care.
Hines et al. (26)	Retrospective cohort study *N* = 3,757,218	B	Good	USA	Primary language was defined by self-report as the language in which the patient preferred to communicate.	No language-based exclusions were made.	Quality, availability, and rates of PMI usage were unknown/not stated.	Preferring a non-English language was not associated with higher stroke mortality in California, with the exception of higher mortality for Japanese speakers.
John-Baptiste et al. (22)	Retrospective cohort study *N* = 44,983	B	Good	Canada	LEP was designated if the patient was unable to communicate in English at admission.	Excluded patients who communicated both in English and a non-English language.	Quality, availability, and rates of PMI usage were unknown/not stated.	LOS was longer for LEP stroke patients (adjusted relative LOS 95% CI 1.18–1.42), but rate of in-hospital death was not significantly different.
Kilkenny et al. (25)	Prospective cohort study *N* = 34,562	B	Good	Australia	Groups were defined based on need for a PMI.	No language-based exclusions were made.	Quality and availability of PMI usage were unknown/not stated.	Patients requiring PMI services had similar discharge outcomes but poorer quality of life 3–6 months after discharge, with significant differences observed within the dimensions of self-care, pain, anxiety or depression, and usual activities.
Rodriguez et al. (16)	Retrospective cohort study *N* = 3,770	B	Good	USA	LEP was defined as speaking English less than “very well” by self-report.	No language-based exclusions were made.	Quality, availability, and rates of PMI usage were unknown/not stated.	LEP patients were more likely to have lower TTR (OR 1.5, 95% CI 1.1–2.2), but were not more likely to be in danger range (defined as INR <1.8 or >3.5).
Rostanski et al. (18)	Retrospective cohort study *N* = 391	B	Good	USA	Primary language was defined by self-report as the language in which the patient preferred to communicate.	No language-based exclusions were made.	Quality, availability, and rates of PMI usage were unknown/not stated.	Spanish speakers were more likely than English speakers to have used EMS, and prenotification rates were not significantly different among those who used EMS. Median onset-to-door and DTN times did not differ between Spanish and English speakers.
Rostanski et al. (20)	Retrospective cohort study *N* = 279	B	Good	USA	Primary language was determined based on self-report.	No language-based exclusions were made.	PMIs available 24/7 via telephone and Spanish in-person interpreters available 24/7 in the ED. Interpretation quality was unknown/not stated.	No differences were found in median DTI time, ITN time, or DTN times between language-concordant and discordant groups.
Rostanski et al. (19)	Cross-sectional study *N* = 350	D	Good	USA	Primary language was determined based on self-report.	No language-based exclusions were made.	PMIs available 24/7 via telephone and Spanish in-person interpreters available 24/7 in the ED. Interpretation quality was unknown/not stated.	The proportion of stroke mimics did not differ between language-concordant and discordant groups, or between English and Spanish speakers.
Shah et al. (23)	Retrospective cohort study *N* = 14,293	B	Good	Canada	Language barrier was defined based on self-reported preferred language.	No language-based exclusions were made.	Quality, availability, and rates of PMI usage were unknown/not stated.	Stroke patients with language barriers had lower 7-day mortality (7.0 vs. 9.2%, OR 0.69, 95% CI 0.57–0.82, *p* <0.001) but were more likely to have a moderate-to-severe neurological deficit at the time of discharge (65.9 vs. 51.5%, OR 1.25, 95% CI 1.15–1.35)
Smith et al. (17)	Prospective cohort study *N* = 1,134	B	Good	USA	Primary language was defined by self-report as the language in which the patient preferred to communicate.	No language-based exclusions were made.	Quality, availability, and rates of PMI usage were unknown/not stated.	Speaking primarily Spanish or English was not associated with time to presentation or mode of arrival in patients with ischemic stroke.
Taylor et al. (30)	Cross-sectional study *N* = 13	D	Poor	UK	Language barriers were defined as any perceived difficulty communicating due to differing language proficiencies.	No language-based exclusions were made.	Quality, availability, and rates of PMI usage were unknown/not stated.	Therapists reported that language barriers affected rehabilitation, implicating causes such as compromised ability to build relationships, provide written material, set goals, assess, treat, and utilize subtleties of communication.
Zachrison et al. (32)	Retrospective cohort study *N* = 3,190	B	Good	USA	Primary language was defined by self-report as the language in which the patient preferred to receive medical information.	Excluded patients who did not indicate a language preference.	Quality, availability, and rates of PMI usage were unknown.	No differences were observed between English-preferring and non-English preferring patients in time from symptom recognition to hospital arrival, rates of arrival by EMS or other mode of transport, DTI time, or DTN time.

*LEP, Limited English proficiency; ADL, activities of daily living; DTI, door-to-imaging; DTN, door-to-needle; EMS, emergency medical services; FIM, Functional Independent Measure; ITN, imaging-to-needle time; LOS, length of stay; NIHSS, National Institutes of Health Stroke Scale; PMI, professional medical interpreter; TTR, time in therapeutic range; TUG, Timed Up and Go test*.

## Results

**Figure 1:**
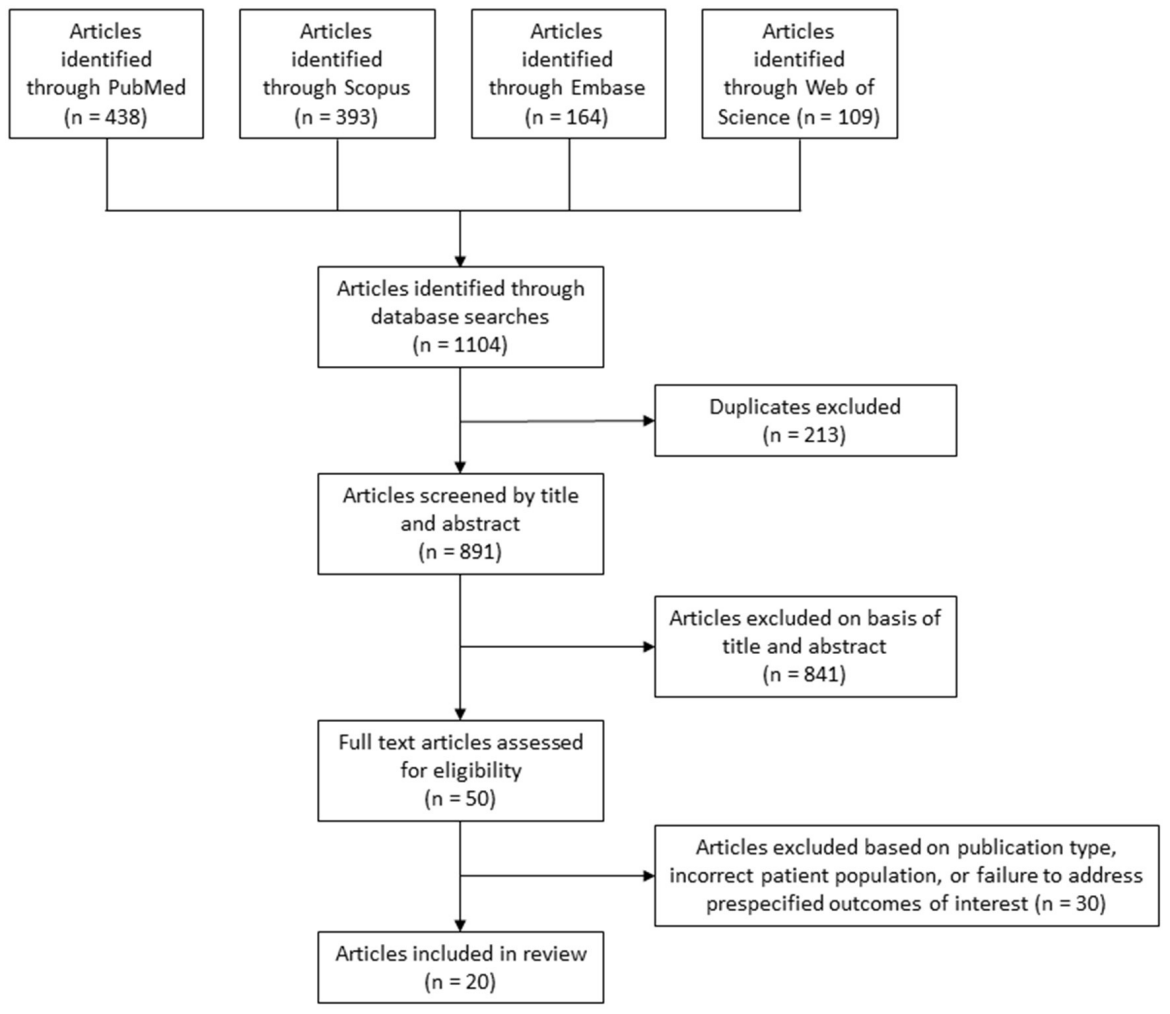
PRISMA flowchart of database search results and article selection process.

Our search strategy identified 891 articles, 20 of which were included in this systematic review (Table 2) (13–32). Our PRISMA flowchart detailing the article selection process is depicted in Figure 1. Eleven retrospective cohort studies were included, as were six cross-sectional studies, two prospective cohort studies, and one retrospective case-control study. Study sample sizes ranged from 13 to 3,757,218 individuals. Seventeen studies were judged to be of good quality based on the Newcastle-Ottawa Quality Assessment, while three studies were of poor quality. Notably, all studies arose from English-predominant countries given our focus on language concordance, including thirteen studies from the USA, four from Australia, two from Canada, and one from the United Kingdom. Four studies examined pre-stroke facets of patient care, namely stroke symptom awareness and preventive treatment, twelve studies examined factors of acute stroke care such as presentation, inpatient management, and outcomes, and five studies investigated aspects of post-stroke care, principally rehabilitation and quality of life. One study investigated both acute care and post-stroke outcomes. No studies excluded individuals with LEP. Nine studies reported details of PMI usage and availability (Table 2). As such, in the eleven studies included in this systematic review which do not provide information on those metrics, it is unknown to what extent an LEP individual in those studies was able to have clear language-concordant interactions with an English-speaking provider, therefore making interpretation of the effect of LEP on measured outcomes in these studies challenging.

### Pre-stroke Care: Awareness and Preventive Treatment

Four studies discussed awareness and preventive treatment prior to stroke (13–16). DuBard et al. showed that after adjusting for sociodemographic characteristics, healthcare access, and cardiovascular risk factors, Spanish-speaking Hispanic respondents were less likely than English-speaking Hispanic, non-Hispanic White, and non-Hispanic Black respondents to correctly identify all stroke symptoms listed in the study's survey (18% of respondents vs. 31, 50, and 41%, respectively, *p* < 0.001) (13). Three studies reported effects of primary language on preventive anticoagulation (14–16). One found that not speaking English was independently associated with describing warfarin indication discordantly with acceptable responses, but not with providing discordant descriptions of stroke (14). Two studies examined differences in mean time in therapeutic range (TTR) for warfarin across groups receiving treatment in specialized anticoagulation clinics. Bhandari et al. reported that in their clinic mean TTR for all patients was 43%, and TTR was 7.2% lower for Spanish-speaking Hispanic patients than for English-speaking Hispanic patients despite intensity of care being indistinguishable across all groups (15). Rodriguez et al. found that mean TTR for all patients combined was 73.8%, however, the LEP population compared to the non-LEP population demonstrated more comorbidities, lower rates of insured status, and lower average level of education (16). Their study also demonstrated that after adjusting for sociodemographic and clinical factors, LEP patients were more likely to have lower TTR (OR 1.5, 95% CI 1.1–2.2), but were not more likely to be in danger range (defined as INR <1.8 or >3.5) (16).

### Acute Stroke Care: Presentation, Inpatient Management, and Outcomes

Twelve studies described acute stroke care (17–26, 31, 32). Two studies found no association between English or non-English preference and either mode of arrival or time to hospital presentation (17, 32). Three studies were carried out at the same New York City institution using data from patients who received IV-tPA (18–20). One found that Spanish speakers were more likely than English speakers to have arrived by EMS after adjusting for confounders, while prenotification rates were not significantly different among those who utilized EMS. Median symptom onset-to-door and DTN times did not differ between Spanish and English speakers (18). A second study investigated the role of language-concordant vs. discordant encounters, defined by whether the primary treating physician and the patient possessed fluency in the same language. The proportion of stroke mimics was not shown to differ between the two groups, nor did it differ between patients who self-reported as primarily English or Spanish speakers (19). The third related study observed no differences in median door-to-imaging (DTI) time, imaging-to-needle (ITN) time, or DTN time between language-concordant and discordant groups (20). Similar results were obtained in two additional studies at different institutions, which showed no differences in DTI and ITN times between English and non-English preferring patients (31, 32), while Anderson et al. demonstrated comparable LOS, functional status at discharge, and mortality between English and non-English-preferring patients (31).

Erfe et al. showed that after adjusting for socioeconomic factors, age, sex, and initial National Institutes of Health Stroke Scale (NIHSS), likelihood of receiving IV thrombolysis did not differ for patients who preferred a language other than English (21). Another study investigated the effect of LEP on LOS and in-hospital mortality for 23 different conditions, finding for stroke patients that while LOS was longer for patients with LEP (adjusted relative LOS 95% CI 1.18–1.42), rate of in-hospital death was not significantly different (22). Shah et al. found that stroke patients with language barriers had lower 7-day mortality (7.0 vs. 9.2%, OR 0.69, 95% CI 0.57–0.82, *p* < 0.001) but were more likely to have a moderate-to-severe neurological deficit at the time of discharge (65.9 vs. 51.5%, OR 1.25, 95% CI 1.15–1.35) (23).

PMI usage was found to be associated with the quality of acute ischemic stroke care. Multivariate analysis accounting for sociodemographic factors and stroke severity showed that non-English-preferring patients who did not receive a PMI were less likely to receive defect-free care than patients who did receive PMI services (61.5 vs. 73.9%, *p* = 0.04, adjusted model OR 0.49, 95% CI 0.25–0.94), where defect-free care represented receipt of all treatment measures for which a patient was eligible, such as thrombolysis within 3 h of symptom onset, antithrombotics prescribed within 48 h of hospitalization, and more (24). Kilkenny et al. showed that patients hospitalized for stroke or transient ischemic attack (TIA) who required interpreters experienced rates of mortality and discharge to rehabilitation that were not distinguishable from patients who did not require interpreters, however, they also had longer LOS and had more often received care on a dedicated stroke unit (85 vs. 78%, *p* < 0.001) (25). Finally, Hines et al. reported higher mortality in Japanese-speaking stroke patients in California, though in general, preferring a non-English language was not associated with higher inpatient mortality (26).

### Post-stroke Care: Rehabilitation and Quality of Life

Five studies examined post-stroke care (25, 27–30). Kilkenny et al. showed that patients who required a PMI had poorer quality of life at 3–6 months post-discharge, with significant differences observed within the dimensions of self-care, pain, anxiety or depression, and usual activities as assessed by the EuroQoL five-dimensions three-level tool (25). Fryer et al. showed that patients returning home following acute stroke rehabilitation who required an interpreter needed more assistance with activities of daily living (ADLs), had lower activity levels and rates of exercise, had slower gait speed and lower functional mobility, and had utilized fewer home health services (27). One study reported that inpatient rehabilitation outcomes and time spent with therapists did not differ between LEP and high English proficiency groups, however, within the LEP group, patients receiving higher levels of PMI services made greater improvements in measures of functional independence (28).

Fryer et al. subsequently interviewed LEP patients about the role of PMIs in their post-stroke care. Patients often saw rehabilitation tasks as tests of competence rather than constructive activities, felt little agency in the decision of whether or not to involve a PMI, and commonly settled for “getting by” in English despite varying levels of proficiency (29). Investigating the other side of such interactions, Taylor et al. interviewed therapists who indicated that rehabilitation was affected by language barriers (30). They identified obstacles including lower frequency of visits due to difficulty logistically arranging PMI services or interpreter unavailability for uncommon languages and dialects, extended duration of sessions due to need for translation, and lower likelihood of providing written materials due to absence of writing translation services. Therapists also reported that their connection with the patient was hindered by reduced or absent informal conversation, and that patient cognition and mood were more challenging to assess without subtleties of language in their interactions. Cognitive communication difficulties, in particular aphasia or dysarthria, were mentioned as specifically challenging to diagnose. Physiotherapists maintained confidence in providing treatment but described feeling less able to assess and treat issues concerning pain and sensation (30).

## Discussion

We present a systematic review focusing on the relationship between LEP and outcomes in stroke care at different stages. Of note, we focused on areas where the primary language utilized in the healthcare system is English given the importance of language concordance for patient-physician communication. We highlight the difficulties faced by LEP individuals in English-speaking healthcare settings, describe the effects of PMI services in stroke patient care, and convey that amidst concerning disparities, high-quality and equitable care is an achievable goal. We also note that the effect of LEP on clinical outcomes in English-speaking environments is not possible to reliably determine without high quality assessment and reporting of the extent and quality of PMI usage. In studies which do not provide information on those metrics, it is unknown to what extent an LEP individual was able to clearly communicate with an English-speaking provider. This contributes to limitations in the ability to draw generalizable conclusions about the effect of LEP on clinical outcomes from these study results and conveys a need for future literature on this topic to report on PMI availability and usage. A better understanding of the impact LEP has on outcomes in stroke may improve resource allocation to enable greater connection to the healthcare system, strengthen the patient-physician relationship, and ultimately improve patient outcomes at all stages of stroke care.

### Pre-stroke Care

Four studies associate LEP with suboptimal results across multiple important metrics of pre-stroke care, showing that LEP individuals have lower awareness of stroke symptoms and experience greater difficulty with medication regimens, reflected by less TTR while undergoing chronic anticoagulation (13–16). Considering the time-sensitive nature of acute stroke management, recognition of stroke symptoms is a crucial early step (33, 34), and preventive anticoagulation serves as an effective defense against stroke especially in those with particular risk factors such as atrial fibrillation or a prosthetic heart valve, emphasizing that disparities in this phase of care place LEP patients at particular risk for poor outcomes (35, 36). Anticoagulation clinics showed poorer results for LEP individuals despite similar intensity of care, indicating that communication and adherence to regimens outside of the clinic may be principal sources of inequity. Stroke symptom awareness and anticoagulation regimen comprehension and adherence may be addressed by patient education considerate of linguistic and cultural diversity as well as of health literacy. Healthcare systems can assist by providing accessible translation services for written information to serve as complements to patient-provider discussions. While utilizing anticoagulant medications requiring less monitoring than warfarin may be a practical strategy to improve TTR in LEP patients, it does not address the systemic nature of this disparity.

### Acute Stroke Care

Acute care appears to be administered with equitable outcomes in a majority, but not all, included studies on this topic. While six studies found no differences in a number of specific metrics of acute care (18–21, 31, 32), Erfe et al. showed that among non-English-preferring patients, those who failed to receive PMI services were half as likely to receive defect-free stroke care (24). This may indicate that while having LEP puts individuals at risk for receiving suboptimal care, the quality of care received may in fact hinge upon the proper implementation of PMI services for LEP patients, a distinction lost by solely categorizing patients based on preferred language without consideration of whether PMIs were utilized. Furthermore, the results obtained by Rostanski et al. may not generalize to acute stroke care of all LEP patients, as their studies examined the effect of speaking nearly exclusively Spanish vs. English within a patient population composed of nearly half of each preferred language group, in a facility with Spanish language PMI services available in the ED (20). These results are encouraging and informative, though they may not fully apply to patients preferring a non-English language that is uncommon and rarely encountered in the population served by the facility. Even so, provision of PMIs in the ED could increase the likelihood that providers are able to communicate reliably with LEP stroke patients with a variety of primary languages.

While inpatient mortality was equivalent between LEP patients and their English-proficient counterparts in three studies (22, 25, 31), this apparent similarity exists despite LEP and PMI-requiring patients being treated more often in dedicated stroke units, and may be further influenced by a potentially higher preference for aggressive care in LEP patients, resulting in improved survival at the cost of greater neurological deficits at discharge (23, 25, 31). Considering that longer LOS was reported for LEP and PMI-requiring patients in three studies, similar mortality rates may not truly signify equitable care or outcomes between these groups (22, 23, 25). Until clarified by future research, additional resources dedicated to care in stroke care units may be a practical step to ensure equitable outcomes for patients with language barriers. Interpreters should also consistently be made available for discussions about goals of care to ensure clarity when deciding on management strategies. As health quality metrics continue to evolve, patient reported outcomes and likelihood to recommend have become an increasing component of assessment of quality of care by third party providers and payors. Improving PMI access for LEP patients may influence these quality metrics in the future.

### Post-stroke Care

Rehabilitation poses challenges for LEP patients, and post-stroke quality of life for this group is lower. Encouragingly, comparable rehabilitation outcomes between LEP and high English proficiency patients were shown to be achievable in a system with in-house PMI services (28). Notably, however, the PMIs in this study received cultural competence training, which may have had a positive impact on the ability of PMIs to communicate clearly with patients. As such, the parity in outcomes in this study may not be directly attributable solely to the language concordance provided by a PMI. The role of cultural competence in patient-provider communication merits further study. A particular predicament appears to be that of LEP patients doing their best to “get by” in English, whether with their own incomplete knowledge, or by relying on surrogate communicators whose knowledge of English may by incomplete (29). While this arrangement may signal to providers that an interpreter is not required because some degree of communication is possible, getting by in English is a suboptimal experience for patients, and educating patients that PMI services are available could empower them to seek interpreter involvement and engage more fully with their care and recovery. Providers should be aware that LEP individuals are at risk of lower post-stroke quality of life, and strategies to communicate with LEP patients should be a consideration in aspects of continuing care. Additional structural changes in routine post-stroke hospitalization practices, including building additional time for patient visits requiring interpreters, will also be necessary to impact outcomes for LEP patients. However, without further research demonstrating value of these structural changes, insurers and hospital systems are unlikely to take on the additional cost burdens.

### Existing Literature and Future Directions

Only nine studies specified the availability of PMI services throughout the patient encounters that they examined, while eleven studies either noted that they lacked the ability to analyze the rates of PMI usage or language concordant vs. discordant encounters, or did not describe these data (Table 2). Examining only the relationship between English proficiency and clinical outcomes without consideration of whether patient encounters were language concordant or discordant with the provider, whether via the provider's multilingual abilities or by the utilization of a PMI, may fail to observe the vulnerability of LEP individuals who do not receive proper PMI services in a language discordant environment. Future studies attempting to compare outcomes should consider both patients' preferred language as well as whether their interactions with providers are language concordant or discordant. Additionally, no studies commented on the quality of interpretation provided. Fundamentally, while PMI services intend to permit clear communication between the provider and patient, some clinical scenarios or concepts may prove to be more challenging to communicate, difficulties which may be compounded by individual patient characteristics such as health literacy, socioeconomic factors, or linguistic requirements such as proficiency in an uncommon language or dialect. Such assessments of interpretation quality may permit detection of more granular differences in clinical outcomes within groups receiving PMI services.

### Limitations

There are several limitations to this systematic review. Only published studies with full text available were included, which places results at risk of publication bias. Studies which showed inequitable outcomes for LEP stroke patients despite PMI implementation may be underrepresented in the literature. The evidence included was generally of good quality, though three articles included were graded as poor quality by the Newcastle-Ottawa Quality Assessment. No randomized trials were available to include, though this topic does not easily lend itself to randomized trial design. Studies originated from a variety of countries and regions, which feature varied demographics, resources, and institutional practices for treating and providing PMIs to LEP patients, meaning that results from individual studies may not be observed in other settings. Similarly, literature from non-English speaking countries was not included in the scope of this systematic review as this would involve excessive heterogeneity in patient populations. However, literature examining language discordance from non-English speaking countries may provide valuable insights into the relationship of patient-provider language discordance with patient outcomes in significantly different sociocultural settings. Even among English-speaking countries alone, sociocultural characteristics are likely to vary substantially within a group of individuals considered to have LEP. For example, differences related to immigration status, education level, health literacy and more are known to be associated with healthcare utilization and outcomes which contributes additional uncertainty as to the generalizability of one set of study results to a different population of LEP individuals (37, 38). Studies defined LEP inconsistently, and in clinical environments the decision of whether to utilize a PMI can be complex and influenced by a variety of factors, such as the nature of the information being shared or acquired, the comfort of the patient or provider with attempting communication in a language in which they are not fluent but in which they may possess some degree of proficiency, the urgency of the situation and availability of or delay in obtaining PMI services, and other considerations. These factors contribute to heterogeneity in the patient populations being studied as well as the true quality of communication in an encounter broadly considered language concordant or discordant. Heterogeneity of study designs, outcomes, and participants precluded performing a meta-analysis. Nevertheless, we provide a comprehensive summary of the effect of LEP on outcomes of stroke prevention, management, and rehabilitation and draw attention to limitations in current research and the need for future studies to take PMI availability and usage into account in order to improve generalizability of results.

## Conclusions

Stroke patients with LEP face barriers to equitable care at multiple stages. Under certain circumstances and with provision of PMIs, equitable care has been demonstrated, if inconsistently, in aspects of stroke prevention and treatment. Patients with LEP may benefit from tailored education regarding stroke symptom recognition and medication regimens. Interpretation services which translate written material will enhance the ability of patients to participate fully in their care and recovery. Studies which categorize patients solely by preferred language may not observe effects of PMI utilization within LEP groups, a factor of stroke care which would benefit from further research, and future studies should report PMI availability and usage within LEP groups in order to allow for more generalizable and reliable conclusions about the effect of LEP and PMI implementation on measured outcomes. All healthcare professionals would benefit from increased awareness of the challenges facing those with LEP and from pursuing quality communication through professional interpreters.

## Data Availability Statement

The original contributions presented in the study are included in the article/Supplementary Material, further inquiries can be directed to the corresponding author/s.

## Author Contributions

JC: conception and design of the work, acquisition, analysis, and interpretation of the data, drafting the manuscript, final approval of the version submitted for publication, and agreement to be accountable for all aspects of the work. NS: conception and design of the work, acquisition, analysis, interpretation of the data, critical revision of the manuscript for intellectual content, final approval of the version submitted for publication, and agreement to be accountable for all aspects of the work. AB and EL: conception and design of the work, critical revision of the manuscript for intellectual content, final approval of the version submitted for publication, and agreement to be accountable for all aspects of the work. All authors contributed to the article and approved the submitted version.

## Funding

Northwestern Open Access Fund provided by Northwestern University Libraries supported the cost of open access publication fees.

## Conflict of Interest

The authors declare that the research was conducted in the absence of any commercial or financial relationships that could be construed as a potential conflict of interest.

## Publisher's Note

All claims expressed in this article are solely those of the authors and do not necessarily represent those of their affiliated organizations, or those of the publisher, the editors and the reviewers. Any product that may be evaluated in this article, or claim that may be made by its manufacturer, is not guaranteed or endorsed by the publisher.
