# Supplementary material for: The Relationship Between Limited English Proficiency and Outcomes in Stroke Prevention, Management, and Rehabilitation: A Systematic Review
Source: Front Neurol. 2022 Feb 3;13:790553. doi: 10.3389/fneur.2022.790553 (PMC8850381; doi:10.3389/fneur.2022.790553)
Supplement: Supplementary file 1 [file Table_1.DOCX]

**Supplemental Table 1: Database Search Strategy**

| **Database** | **Search Terms** |
| --- | --- |
| PubMed | (stroke) AND ((Limited English Proficiency OR Limited English Proficient) OR (language* AND "english" AND ("translator*" OR "interpret*" OR "language barrier*" OR "communication barrier*" OR "language discord*" OR "language interpret*" OR "patient language" OR "english speak*" OR "language preference"))) |
| Embase | stroke:ti,ab,kw AND ('limited english proficiency':ti,ab,kw OR 'limited english proficient':ti,ab,kw OR (language*:ti,ab,kw AND 'english':ti,ab,kw AND ('translator*':ti,ab,kw OR 'interpret*':ti,ab,kw OR 'language barrier*':ti,ab,kw OR 'communication barrier*':ti,ab,kw OR 'language discord*':ti,ab,kw OR 'language interpret*':ti,ab,kw OR 'patient language':ti,ab,kw OR 'english speak*':ti,ab,kw OR 'language preference':ti,ab,kw))) |
| Scopus | TITLE-ABS-KEY(stroke) AND (Limited English Proficiency OR Proficient OR (language* AND "english" AND ("translator*" OR "interpret*" OR "language barrier*" OR "communication barrier*" OR "language discord*" OR "language interpret*" OR "patient language" OR "english speak*" OR "language preference"))) |
| Web of Science | TS=((stroke) AND ((Limited English Proficiency OR Limited English Proficient) OR (language* AND "english" AND ("translator*" OR "interpret*" OR "language barrier*" OR "communication barrier*" OR "language discord*" OR "language interpret*" OR "patient language" OR "english speak*" OR "language preference")))) |
